# The Unprecedented Movement Control Order (Lockdown) and Factors Associated With the Negative Emotional Symptoms, Happiness, and Work-Life Balance of Malaysian University Students During the Coronavirus Disease (COVID-19) Pandemic

**DOI:** 10.3389/fpsyt.2020.566221

**Published:** 2021-02-16

**Authors:** Wan Mohd Azam Wan Mohd Yunus, Siti Khadijah Zainal Badri, Siti Aisyah Panatik, Firdaus Mukhtar

**Affiliations:** ^1^Department of Psychology, School of Human Resource Development and Psychology, Faculty of Social Sciences and Humanities, Universiti Teknologi Malaysia, Skudai, Malaysia; ^2^Research Centre for Child Psychiatry, University of Turku, Turku, Finland; ^3^Division of Organisational and Applied Psychology, Business School, Faculty of Arts and Social Sciences, University of Nottingham, Semenyih, Malaysia; ^4^Department of Psychiatry, Faculty of Medicine and Health Sciences, Universiti Putra Malaysia, Serdang, Malaysia

**Keywords:** anxiety, depression, students, lockdown, COVID-19 pandemic, stress, happiness, work-life balance

## Abstract

**Background and Aims:** Malaysia's first Movement Control Order (MCO) or “lockdown” was in place for 6 weeks to curb the spread of coronavirus disease (COVID-19). Consequently, all universities were forced to close temporarily with abrupt changes to teaching and learning activities. However, there has been a lack of consensus regarding students' actual psychological status and mental health during the MCO implementation. This study investigates the link, state, and differences of negative emotional symptoms, happiness, and work-life balance among university students during the COVID-19 pandemic.

**Methodology:** This study recruited 1,005 university students across Malaysia. Data was collected online using Qualtrics to measure negative emotional symptoms (The Depression, Anxiety, and Stress Scale), happiness (The Oxford Happiness Inventory), and work-life balance (Work-Family Conflict Scale). All data was analyzed using SPSS version 25 and AMOS version 26 using *T*-test, ANOVA, logistic regression analyses, and path analysis method.

**Findings:** Findings indicated that 22, 34.3, and 37.3% of the university students scored moderate to extremely severe levels of stress, anxiety, and depression symptoms, respectively. Half scored rather happy or very happy (50%) for happiness levels. Meanwhile, 50.4 and 39.4% scored high to very high levels of work-to-family and family-to-work conflict. Significant differences in stress, anxiety, depression, happiness, work-family conflict, and family-work conflict were recorded across different demographic factors. Happiness was found to be a protective factor with a lesser likelihood of experiencing severe stress (OR = 0.240, 95% CI: 0.180, 0.321), anxiety (OR = 0.336, 95% CI: 0.273, 0.414), and depression (OR = 0.121, 95% CI: 0.088, 0.165) with higher happiness levels. Higher score of work-to-family conflict contributes to greater odds of having severe levels of anxiety (OR = 1.453, 95% CI: 1.161, 1.818). While greater likelihood of developing severe stress (OR = 1.468, 95% CI: 1.109, 1.943) and severe anxiety (OR = 1.317, 95% CI: 1.059, 1.638) under increasing score of family-to-work conflict. Besides, happiness is found to negatively linked with lower negative emotional symptoms, while work-family conflict and family-work conflict are positively linked with higher negative emotional symptoms.

**Conclusion:** Lockdown implementation during the COVID-19 pandemic appears to have a significant impact on university students' negative emotional symptoms, happiness, and work-life balance. Happiness was found to be a protective factor while the state of work-life balance is a risk factor that can predict students' negative emotional symptoms.

## Introduction

On 11 March 2020, the World Health Organization declared the coronavirus disease (COVID-19) a global pandemic resulting in many countries worldwide having to enforce unprecedented lockdown measures to curb the spread of novel virus severe acute respiratory syndrome coronavirus 2 (SARS-CoV-2). As of 31st December 2020, the virus has killed more than 1.8 million people worldwide, causing spiking fears, anxiety, and state of panic which result to various well-being and socioeconomic concerns among human population (1). Due to its substantial impact worldwide, new research on the psychological, behavioral, interpersonal, and clinical implications toward high-risk population and different contexts are imperative (2). On 25th January 2020, Malaysia had the first three positive cases of COVID-19. On 16th March 2020, Malaysia's Prime Minister has officially announced the unprecedented Movement Controlled Order (MCO), in response to the pandemic. The order promulgates restriction of social activities under the Prevention and Control of Infectious Diseases Act 1988 and the Police Act 1967. During the MCO period, only essential businesses were allowed to operate while most service sectors including schools and higher institutions suspended all physical activities. Concurrent with the steady increase of daily cases, the full MCO implementation has been extended twice, accumulating to a total of 6 weeks since the 1st date of enforcement has taken place.

A rapid review of previous outbreaks suggests that a state of isolation or restricted access measures has severe effects on individual psychological condition such as posttraumatic stress symptoms, confusion, and anger caused by various conditions such as fears of infection, frustration, boredom, inadequate supplies or information, financial loss, and stigma (3). Likewise, recent studies during the COVID-19 pandemic have also provide evidences of indirect adverse mental health impact of the virus (4, 5). Early evidence suggests that the impact of COVID-19 is beyond massive contraction of global economic activities, similarly pointing to the degrading quality in health and well-being (6). While this will leave no sector in any country unaffected, the socioeconomic impact in all education sectors from preschool to tertiary education has been reported (7).

Young adults and educated individuals are particularly found to be more vulnerable to mental distress based on recent studies (8, 9). In addition, the conditions of disrupted daily life and delays in academic activities were positively linked to the declining of students' mental health condition (10), rising unhappiness, and conflict (11). However, despite serious attention given to this topic, there has been a lack of consensus drawing on university student population with existing data skewed toward certain countries. The unprecedented period of pandemic and restriction order has posed a new challenge toward universities. Many universities took the approach to resume academic activities under remote or online teaching methods while those with lacking resources or preparedness were advised to delay the semester. Studies from previous outbreaks have shown that university students are vulnerable to psychological problems (12–14). There are various factors contributing to the deterioration of student psychological well-being in universities such as fear of dormitory evacuation, event cancellation such as study exchange or uncertainty to graduate and fear of losing future job (15, 16). In addition, factors such as major changes in students' routine and daily life, delayed academic activities, and concern over financial struggle and economic fluctuation were also mentioned which overall might exacerbate the students' psychological condition (10).

Given the sudden consequences (such as online teaching or last-minute change in assessment method) this population is facing in complying with the pandemic control efforts, there is a need to investigate the extent to which the state of the pandemic has affected their negative emotional symptoms, happiness, and work-life balance. This study aims to achieve three objectives (a) to identify the state of negative emotional symptoms, happiness, and work-life balance among university students during the second and third MCO phases; (b) to compare the state of negative emotional symptoms, happiness, and work-life balance among university students during the second and third MCO phases based on demographic factors; and (c) to investigate the risk and protective factors that can predict the state of negative emotional systems among Malaysian university students. This will serve as an important evidence to inform educational policy and future health promotion interventions for the university students in Malaysia.

## Materials and Methods

This study utilized a cross-sectional design using Qualtrics online survey to collect data. Samples were recruited among public and private university students in Malaysia using snowball sampling. Data collection took place within 9 days from 15th April 2020 and ended on 23rd April 2020 when Malaysia was at its second and third phases of MCO. A total of 1,458 responses were retrieved; however, 453 responses were found incomplete making a final 1,005 usable responses. A link to the survey invitation was sent and shared through various social media platforms such as Facebook, WhatsApp, Twitter, and e-mail invitations. The survey was designed using dual languages of Bahasa Malaysia and English. Students in any public or private universities in Malaysia at the time of the study and with no history of psychiatric/mental disorders were eligible to participate.

### Ethical Consideration and Data Collection Procedure

This study has received an ethical evaluation from the Faculty of Arts and Social Sciences, University of Nottingham Malaysia Campus ethics committee (Approval number: FASS2020-0008/DOAP/SKH). A consent form was included in the first page of the survey informing the nature of study, voluntary, and withdrawal terms.

The link or QR code directed potential participants to the research information page and the next consent page. This consent form made clear the commitments and expectations relating to the project (commitments to anonymity and confidentiality, details about uses of data, etc.). The consent form page emphasized a range of issues for which consent was sought, and each participant was asked to indicate their support for each one. All participants were also asked to provide explicit consent by typing “I AGREE” or “SAYA SETUJU.”

### Instruments

*Depression, Anxiety, and Stress Scale-21 (DASS-21)* (17). Negative emotional symptoms were assessed using 21-item DASS-21. It consists of depression, anxiety, and stress dimensions with seven items representing each. All items were rated using the scale of 0 to 4—0 (did not apply to me at all), (1) applied to me to some degree or some of the time, (2) applied to me to a considerable degree or a good part of the time, and (3) applied to me very much, or most of the time. Score was calculated using total score multiplied by 2 and further categorized into normal, mild, moderate, severe, or extremely severe levels. Our analyses showed that this scale has good internal consistency using Cronbach's alpha. The Malay version of DASS-21 scored 0.951 while the English version scored 0.940. The DASS-21 has also been used in previous studies during the COVID-19 pandemic (4, 5, 18).

*Oxford Happiness Questionnaire (OHQ)* (19). The 29-item OHQ was used to measure students' happiness. All items were rated using a 6-point Likert scale ranging from 1 (strongly disagree) to 6 (strongly agree). Our analysis recorded good Cronbach's alpha value with the Malay version scoring 0.723 while the English version scoring 0.827.

*Work-family Conflict Scale (WFC)* (20). Work-life balance was measured using a 10-items WFC scale consisting of two subdimensions of work-to-family and family-to-work conflict. The scale was primarily developed for working population. However, as this juncture shows no relevant scale to measure university student's work-related activities (assignment, logbook, lab work, etc.), this scale is therefore used by explaining to the respondents to relate the items with their university's work. For this scale, the Cronbach's alpha is also good with the Malay version recording 0.932 while the English version scored 0.946.

### Statistical Analysis

Data analysis was performed using IBM Statistical Package for Social Science (SPSS) version 25 and AMOS version 26 employing both descriptive and inferential statistics. *T*-test and ANOVA analysis were performed for comparing means between groups according to demographic factors. Meanwhile, multivariate logistic regression analysis was performed to assess potential risk and protective factors for the incidence of severe depression, anxiety, and stress symptoms among participants. Results were presented using odds ratios (ORs) and bias corrected (BC) 95% confidence intervals. Path analysis was performed to examine the link between constructs of the study by observing beta estimates (β).

### Data Assumptions

Multicollinearity and normality assumptions were assessed using skewness, kurtosis, and *Q*–*Q* plot. Following the suggestion by Garson (21), data was meeting the range for acceptable with skewness and kurtosis with ±2 for skewness and ±3 for kurtosis with a straight diagonal line for *Q*–*Q* plot observed.

## Results

### Demographic Profile, Location, and Status at the Period of Lockdown

Table 1 presents the descriptive characteristics of our participants. In summary, the majority of the respondents were female with 75.5% (*n* = 759), while male participants only made up a total of 24.5% (*n* = 246) from the entire population. Most respondents (*n* = 523) were between 17 and 22 years old. Only 6% were aged 35 years and above (*n* = 60). Results indicated Malay ethnicity representing almost a three-quarter (*n* = 746, 74.3%) of the overall population in this study. Public university students recorded 70.2% (*n* = 706), meanwhile only 29.8% were from private universities (*n* = 299).

**Table 1:** Respondents' demographic characteristics (*N* = 1,005).

**Category**	***F***	**%**	**Std**	**Skewness**	**Kurtosis**
Gender	1,005	100	0.014	−1.189	−0.587
Male	246	24.5			
Female	759	75.5			
Age	1,005	100	0.029	1.883	2.233
17–22 years old	523	52.0			
23–28 years old	376	37.4			
29–34 years old	45	4.5			
35 years old and above	60	6.0			
Ethnicity	1,005	100	0.029	0.077	2.233
Malay	746	74.3			
Chinese	128	12.7			
Indian	47	4.7			
Others	84	8.4			
University	1,005	100	0.014	0.917	−1.162
Public	706	70.2			
Private	299	29.8			
Location at the period of MCO/pandemic	1,005	100	0.045	0.177	−1.355
Central region	362	36.0			
East coast region	112	11.1			
Northern region	139	13.8			
Southern region	327	32.5			
East Malaysia	57	5.7			
Outside Malaysia	8	0.80			
Level of study	1,005	100	0.746	1.947	4.689
Diploma	69	6.9			
Bachelor's degree	774	77.0			
Master's degree	85	8.5			
Doctoral degree	50	5.0			
Field of study	1,005	100	0.514	−0.164	−1.270
Technical	401	39.9			
Non-technical	590	58.7			
Not specified	14	1.4			
Status of resident during MCO/pandemic	1,005	100	0.824	−0.717	−0.262
Staying at hometown	603	60.0			
Staying in-campus	151	15.0			
Staying outside-campus	183	18.2			
Others	68	6.8			
Risk to contagion	1,005	100	0.539	2.239	3.937
High	825	82.1			
Moderate	124	12.3			
Low	56	5.6			

*f, frequency; %, percentage; std, standard deviation error*.

At the point of data collection, a total of 36.0% of our respondents were located in the central region (*n* = 362), 11.1% in the east coast (*n* = 112), a total of 13.8% were located in the northern region (*n* = 139), following 32.5% in the southern region (*n* = 327). Those in East Malaysia and outside Malaysia were represented by 5.7% (*n* = 57) and 0.8% (*n* = 8), respectively. Information for the study program was reclustered into two categories of technical and non-technical programs as defined by the Ministry of Higher Education Malaysia (22). For this, a total of 39.9% were from technical programs (*n* = 401), meanwhile, 58.7% (*n* = 590) were enrolled in non-technical programs. A very small percentage of 1.4% chose not to state their program. Lastly, more than half of our respondents (*n* = 603, 60%) were currently at their hometown, 15.0% (*n* = 151) were on campus, meanwhile 18.2 stayed outside campus (*n* = 183).

### Students Levels of Negative Emotional Symptoms, Happiness, and Work-Family Conflict During the Period of COVID-19 Lockdown

Tables 2–**4** summarize negative emotional symptoms, happiness, and work-family conflict levels among our respondents. Descriptive statistics were analyzed using mean and frequency in the SPSS. In summary, the result suggests that the majority of students scored normal to mild for stress, anxiety, and depression levels. A total of 7.9, 8.0, and 8.4% scored severe stress, anxiety, and depression levels. This was followed by 2.8, 14.8, and 10.6% who scored extremely severe stress, anxiety, and depression levels (please refer to Table 2). Combining moderate to extremely severe levels altogether, this study found a total percentage of 22.0% of students suffering from stress, 34.3% from anxiety, and 37.3% from depression symptoms.

**Table 2:** Stress, anxiety and depression symptoms severity categorization (*N* = 1,005).

	**Normal**		**Mild**		**Moderate**		**Severe**		**Extremely severe**	
	***f***	**%**	***f***	**%**	***f***	**%**	***f***	**%**	***f***	**%**
DASS-S	664	66.1	120	11.9	114	11.3	79	7.9	28	2.8
DASS-A	498	49.6	162	16.1	116	11.5	80	8.0	149	14.8
DASS-D	487	48.5	143	14.2	184	18.3	84	8.4	107	10.6

*f, frequency; %, percentage; DASS-S, stress; DASS-A, anxiety; DASS-D, depression*.

a *Scoring is based on a manual published by (23). After multiplying the scores by 2, the total score was categorized into five; normal, mild, moderate, severe, or extremely severe. Depression was scored with a total score from 0 to 9 for normal, 10 to 13 for mild, 14 to 20 for moderate, 21 to 27 for severe depression, and 28 to 42 for extremely severe depression. Anxiety was calculated based on scores 0–7 normal, 8–9 for mild, 10–14 for moderate, 15–19 for severe, and 20–42 for extremely severe. Lastly, stress was scored from 0 to 14 for normal, 15 to 18 for mild, 19 to 25 for moderate, 26 to 33 for severe, and 34 to 42 for extremely severe*.

Table 3 indicated that half of the participating students scored from “rather happy” and “very happy” for happiness. Few students accounted for 11.6% indicating that they were unhappy or somewhat unhappy, 38.4% of our respondents were not particularly happy or unhappy making a total of 50.0% for these three categories.

**Table 3:** Happiness total score categorization (*N* = 1,005).

	**Not happy**		**Somewhat unhappy**		**Not particularly happy or unhappy**		**Rather happy**		**Very happy**	
	***f***	**%**	***f***	**%**	***f***	**%**	***f***	**%**	***f***	**%**
Happiness OHQ	9	0.90	108	10.7	386	38.4	421	41.9	81	8.1

*Not happy score is between 0 and 58, somewhat happy score is between 59 and 87, not particularly happy or unhappy score is between 88 and 116, rather happy is between 117 and 145, very happy score is between 146 and 173, and too happy score is 174*.

*f, frequency; %, percentage*.

Meanwhile, results in Table 4 suggested that the majority of students were experiencing low to moderate levels of work-to-family conflict and family-to-work. Those who experienced moderate to extremely high levels of work-to-family conflict accounted for 50.4% from the overall percentage for work-to-family conflict and 39.2% for family-to-work conflict.

**Table 4:** Work-family conflict and family-work conflict total score categorization (*N* = 1,005).

	**Very low**		**Low**		**Moderate^a^**		**High^a^**		**Extremely high^a^**	
	***f***	**%**	***f***	**%**	***f***	**%**	***f***	**%**	***f***	**%**
W-FC	102	10.1	396	39.4	302	30.0	148	14.7	57	5.7
F-WC	173	17.2	438	43.6	222	22.1	125	12.4	47	4.7

*f, frequency; %, percentage; W-FC, work-to-family conflict; F-WC, family-to-work conflict*.

a *Moderate-extremely high defined as W-FC ≥ 16, F-WC ≥ 16. Scoring: 0–5 for very low, 6–10 for low, 11–15 for moderate, 16–20 for high, and 21–25 for very high*.

### Differences in Students' Level of Negative Emotional Symptoms, Health, Happiness, and Work-Family Conflict Based on Demographic Factors

*T*-test and one-way ANOVA were performed to assess differences in students' level of negative emotional symptoms, happiness, and work-family conflict during the lockdown period based on demographic profiles. Overall test and *post*-*hoc* results in Tables 5, 6 revealed that there were significant differences in the DASS-S, DASS-A, DASS-D, happiness, W-FC, and F-WC levels across different demographic factors. Detailed results indicated differences in terms of DASS-S mean level for age, location, university type, and ethnicity with the *p* < 0.05. Specifically, students from the northern region (μ = 1.679, *p* < 0.001) had lower stress symptoms compared with those in the East Coast (μ = 1.936, *p* < 0.001), central regions (μ = 1.917, *p* < 0.001), and outside the country (μ = 2.298, *p* < 0.001).

**Table 5:** Demographic characteristics by the DASS-S, DASS-A, DASS-D, OHQ, and WFC.

**Variable**	**DASS-S**				**DASS-A**				**DASS-D**				**Happiness**				**W-FC**				**F-WC**			
	**M**	**±**	**SD**	***p***	**M**	**±**	**SD**	***p***	**M**	**±**	**SD**	***p***	**M**	**±**	**SD**	***p***	**M**	**±**	**SD**	***p***	**M**	**±**	**SD**	***p***
**Gender**				0.748				0.276				0.418				0.370				0.443				0.826
Female	1.818	±	0.687		1.572	±	0.592		1.902	±	0.760		4.146	±	0.937		2.333	±	1.005		2.128	±	1.011	
Male	1.853	±	0.658		1.642	±	0.631		1.911	±	0.731		4.183	±	0.890		2.315	±	0.995		2.121	±	1.012	
**Age**				**0.012**				**0.000**				**0.000**				**0.000**				0.157				0.224
17–22	1.873	±	0.661		1.667	±	0.643		1.984	±	0.732		4.102	±	0.959		2.338	±	1.028		2.162	±	1.034	
23–28	1.858	±	0.660		1.624	±	0.616		1.898	±	0.753		4.172	±	0.887		2.298	±	0.963		2.095	±	0.973	
29–34	1.633	±	0.663		1.431	±	0.526		1.637	±	0.673		4.508	±	0.847		2.044	±	0.809		1.853	±	0.988	
35>	1.647	±	0.647		1.377	±	0.415		1.500	±	0.543		4.590	±	0.955		2.473	±	1.028		2.167	±	1.053	
**Location**				**0.000**				**0.000**				**0.000**				**0.001**				**0.004**				**0.002**
Central	1.917	±	0.679		1.667	±	0.656		1.989	±	0.748		4.024	±	0.884		2.239	±	0.947		2.059	±	0.959	
Northern	1.679	±	0.601		1.437	±	0.520		1.736	±	0.681		4.354	±	0.907		2.137	±	0.946		1.961	±	1.050	
East Coast	1.936	±	0.655		1.786	±	0.663		1.937	±	0.627		4.174	±	0.823		2.463	±	0.992		2.321	±	1.064	
Southern	1.808	±	0.642		1.639	±	0.613		1.910	±	0.769		4.229	±	0.899		2.417	±	1.046		2.208	±	1.016	
East Malaysia	1.713	±	0.682		1.386	±	0.439		1.681	±	0.704		4.383	±	1.036		2.284	±	0.933		1.891	±	0.939	
**University type**				**0.020**				**0.001**				0.466				0.096				**0.044**				0.275
Public	1.801	±	0.641		1.609	±	0.598		1.887	±	0.731		4.239	±	0.909		2.377	±	1.017		2.154		1.014	
Private	1.962	±	0.708		1.676	±	0.679		1.976	±	0.753		3.998	±	0.863		2.173	±	0.933		2.043		1.000	
**Residence**				0.369				0.350				0.306				0.802				0.390				0.490
In campus	1.895	±	0.695		1.669	±	0.661		2.031	±	0.743		4.095	±	0.940		2.238	±	0.973		2.062	±	1.013	
Outside campus	1.905	±	0.698		1.604	±	0.634		1.967	±	0.789		4.112	±	0.875		2.411	±	1.006		2.222	±	1.020	
Hometown	1.818	±	0.651		1.620	±	0.610		1.865	±	0.722		4.203	±	0.896		2.326	±	1.009		2.108	±	1.019	
Others	1.801	±	0.616		1.624	±	0.616		1.863	±	0.699		4.261	±	0.929		2.197	±	0.910		2.115	±	0.912	
**Study level**				0.304				0.091				0.799				0.129				0.625				0.156
Diploma	1.838	±	0.648		1.641	±	0.610		1.928	±	0.742		4.144	±	0.894		2.174	±	0.944		1.980	±	1.027	
Bachelor	1.854	±	0.664		1.646	±	0.642		1.928	±	0.733		4.381	±	0.912		2.334	±	1.012		2.149	±	1.022	
Master	1.825	±	0.672		1.511	±	0.522		1.798	±	0.796		4.325	±	0.946		2.313	±	0.899		2.002	±	0.938	
Ph.D.	1.733	±	0.670		1.464	±	0.509		1.670	±	0.597		3.990	±	0.841		2.388	±	1.065		2.276	±	1.067	
Others	1.870	±	0.709		1.630	±	0.498		2.080	±	0.847		4.144	±	0.894		2.156	±	0.865		1.980	±	0.664	
**Risk to contagion**				0.277				**0.030**				0.680				0.162				0.651				0.459
High	1.835	±	0.668		1.608	±	0.619		1.907	±	0.743		4.152	±	0.914		2.303	±	0.988		2.101		0.995	
Moderate	1.909	±	0.626		1.765	±	0.654		1.920	±	0.697		4.254	±	0.821		2.389	±	1.012		2.225		1.043	
Low	1.741	±	0.570		1.561	±	0.580		1.821	±	0.725		4.356	±	0.868		2.271	±	0.964		2.082		1.122	
**Field of study**				0.296				0.806				0.128				0.081				0.219				0.304
Technical	1.873	±	0.696		1.618	±	0.643		1.955	±	0.768		4.120	±	0.916		2.310	±	1.018		2.118		0.994	
Non-technical	1.831	±	0.642		1.631	±	0.609		1.883	±	0.718		4.201	±	0.893		2.337	±	0.987		2.135		1.026	
Not specified	1.631	±	0.680		1.529	±	0.631		1.643	±	0.606		4.594	±	0.729		1.871	±	0.687		1.714		0.825	
**Ethnicity**				**0.002**				0.313				0.209				**0.000**				**0.004**				**0.008**
Malay	1.827	±	0.647		1.666	±	0.631		1.914	±	0.737		2.275	±	0.941		2.376	±	1.009		2.173		1.030	
Chinese	1.859	±	0.708		1.433	±	0.554		1.823	±	0.705		2.162	±	0.875		2.245	±	0.951		2.078		0.971	
Indian	1.830	±	0.616		1.455	±	0.495		1.823	±	0.702		1.811	±	0.721		1.915	±	0.806		1.706		0.725	
Others	1.988	±	0.762		1.638	±	0.643		2.042	±	0.800		2.064	±	0.931		2.152	±	0.989		1.976		0.982	

*All significant results are marked with bold text (p < 0.05)*.

**Table 6:** The results of *post*-*hoc* analyses (*n* = 1,005).

**Variable**		**DASS-S**		**DASS-A**		**DASS-D**		**Happiness**		**W-FC**		**F-WC**	
**I**	**J**	**M difference (I–J)**	***p***	**Mean difference (I–J)**	***P***	**Mean difference (I–J)**	***p***	**Mean difference (I–J)**	***p***	**Mean difference (I–J)**	***p***	**Mean difference (I–J)**	***p***
**Age**													
17–22 years old	23–28 years old	0.015	0.987	0.043	0.731	0.087	0.291	−0.070	0.656	–	–	–	–
	29–34 years old	0.240	0.091	0.236	0.067	0.347^*^	0.012	−0.406^*^	0.018	–	–	–	–
	35>	0.226	0.059	0.290^*^	0.003	0.484^*^	0.000	−0.488^*^	0.000	–	–	–	–
23–28 years old	17–22 years old	−0.015	0.987	−0.043	0.731	−0.087	0.291	−0.070	0.656	–	–	–	–
	29–34 years old	0.224	0.137	0.193	0.195	0.261	0.106	−0.406^*^	0.018	–	–	–	–
	35>	0.210	0.100	0.247^*^	0.021	0.398^*^	0.001	−0.488^*^	0.000	–	–	–	–
29–34 years old	17–22 years old	−0.240	0.091	−0.236	0.067	−0.347^*^	0.012	0.070	0.656	–	–	–	–
	23–28 years old	−0.224	0.137	−0.193	0.195	−0.261	0.106	−0.336	0.080	–	–	–	–
	35>	−0.014	1.000	0.054	0.970	0.137	0.775	−0.419^*^	0.004	–	–	–	–
35>	17–22 years old	−0.226	0.059	−0.290^*^	0.003	−0.484^*^	0.000	0.406^*^	0.018	–	–	–	–
	23–28 years old	−0.210	0.100	−0.247^*^	0.021	−0.398^*^	0.001	0.336	0.080	–	–	–	–
	29–34 years old	0.014	1.000	−0.054	0.970	−0.137	0.775	−0.083	0.966	–	–	–	–
**Location (region)**													
Central	Northern	0.238^*^	0.004	0.230^*^	0.003	0.253^*^	0.008	−0.330^*^	0.003	–	–	–	–
	East Coast	−0.019	1.000	−0.118	0.481	0.051	0.987	−0.150	0.634	–	–	–	–
	Southern	0.109	0.259	0.028	0.991	0.079	0.722	−0.205^*^	0.033	–	–	–	–
	East Malaysia	0.204	0.253	0.281^*^	0.017	0.307^*^	0.039	−0.359	0.056	–	–	–	–
	Outside	−0.380	0.277	−0.061	0.999	−0.249	0.812	−0.025	1.000	–	–	–	–
Northern	**Central**	−0.238^*^	0.004	−0.230^*^	0.003	−0.253^*^	0.008	0.330^*^	0.003	–	–	–	–
	**East Coast**	−0.257^*^	0.026	−0.348^*^	0.000	−0.201	0.256	0.180	0.608	–	–	–	–
	Southern	−0.130	0.374	−0.202^*^	0.015	−0.174	0.179	0.125	0.741	–	–	–	–
	East Malaysia	−0.035	0.999	0.051	0.995	0.055	0.997	−0.029	1.000	–	–	–	–
	Outside	−0.619^*^	0.011	−0.291	0.538	−0.502	0.142	0.305	0.829	–	–	–	–
East Coast	Central	0.019	1.000	0.118	0.481	−0.051	0.987	0.150	0.634	–	–	–	–
	Northern	0.257^*^	0.026	0.348^*^	0.000	0.201	0.256	−0.180	0.608	–	–	–	–
	Southern	0.128	0.485	0.147	0.248	0.028	0.999	−0.055	0.993	–	–	–	–
	East Malaysia	0.223	0.299	0.400^*^	0.001	0.256	0.263	−0.209	0.704	–	–	–	–
	Outside	−0.362	0.379	0.057	0.999	−0.301	0.698	0.125	0.996	–	–	–	–
Southern	Central	−0.109	0.259	−0.028	0.991	−0.079	0.722	0.205^*^	0.033	–	–	–	–
	Northern	0.130	0.374	0.202^*^	0.015	0.174	0.179	−0.125	0.741	–	–	–	–
	East Coast	−0.128	0.485	−0.147	0.248	−0.028	0.999	0.055	0.993	–	–	–	–
	East Malaysia	0.095	0.916	0.253	0.048	0.228	0.251	−0.154	0.838	–	–	–	–
	Outside	−0.489	0.071	−0.089	0.995	−0.328	0.571	0.180	0.977	–	–	–	–
East Malaysia	Central	−0.204	0.253	−0.281^*^	0.017	−0.307^*^	0.039	0.359	0.056	–	–	–	–
	Northern	0.035	0.999	−0.051	0.995	−0.055	0.997	0.029	1.000	–	–	–	–
	East Coast	−0.223	0.299	−0.400^*^	0.001	−0.256	0.263	0.209	0.704	–	–	–	–
	Southern	−0.095	0.916	−0.253^*^	0.048	−0.228	0.251	0.154	0.838	–	–	–	–
	Outside Malaysia	−0.584^*^	0.035	−0.343	0.422	−0.557	0.111	0.334	0.811	–	–	–	–
Outside country	Central	0.380	0.277	0.061	0.999	0.249	0.812	0.025	1.000	–	–	–	–
	Northern	0.619^*^	0.011	0.291	0.538	0.502	0.142	−0.305	0.829	–	–	–	–
	East Coast	0.362	0.379	−0.057	0.999	0.301	0.698	−0.125	0.996	–	–	–	–
	Southern	0.489	0.071	0.089	0.995	0.328	0.571	−0.180	0.977	–	–	–	–
	East Malaysia	0.584^*^	0.035	0.343	0.422	0.557	0.111	−0.334	0.811	–	–	–	–
**Risk of contagion**													
High risk	Moderate risk	–	–	−0.157^*^	0.031	–	–	–	–	–	–	–	–
	Low risk	–	–	0.047	0.848	–	–	–	–	–	–	–	–
Moderate risk	High risk	–	–	0.157^*^	0.031	–	–	–	–	–	–	–	–
	Low risk	–	–	0.204	0.109	–	–	–	–	–	–	–	–
Low risk	High risk	–	–	−0.047	0.848	–	–	–	–	–	–	–	–
	Moderate risk	–	–	−0.204	0.109	–	–	–	–	–	–	–	–
**Ethnicity**												–	–
Malay	Chinese	–	–	0.234^*^	0.000	–	–	–	–	0.131	0.511	0.095	0.759
	Indian	–	–	0.211	0.105	–	–	–	–	0.462^*^	0.011	0.467^*^	0.011
	Others	–	–	0.028	0.978	–	–	–	–	0.224	0.203	0.197	0.325
Chinese	Malay	–	–	−0.234^*^	0.000	–	–	–	–	−0.131	0.511	−0.095	0.759
	Indian	–	–	−0.023	0.997	–	–	–	–	0.330	0.207	0.372	0.134
	Others	–	–	−0.205	0.084	–	–	–	–	0.093	0.909	0.102	0.889
Indian	Malay	–	–	−0.211	0.105	–	–	–	–	−0.462^*^	0.011	−0.467^*^	0.011
	Chinese	–	–	0.023	0.997	–	–	–	–	−0.330	0.207	−0.372	0.134
	Others	–	–	−0.183	0.365	–	–	–	–	−0.237	0.554	−0.270	0.455
Others	Malay	–	–	−0.028	0.978	–	–	–	–	−0.224	0.203	−0.197	0.325
	Chinese	–	–	0.205	0.084	–	–	–	–	−0.093	0.909	−0.102	0.889
	Indian	–	–	0.183	0.365	–	–	–	–	0.237	0.554	0.270	0.455

* *Only significant result (p < 0.05) are presented. Variables with no significant result are not presented in this table or marked with “–”*.

For DASS-A, findings indicated that a total of four demographic factors were significant. In detail, private university students (μ = 1.676, *p* < 0.001) generally had a higher anxiety level compared with the public university students (μ = 1.609, *p* < 0.001). Younger students within the age bracket between 17 and 22 years old (μ = 1.667, *p* < 0.001) and 23 and 28 years old (μ = 1.624, *p* < 0.001) had higher symptoms of anxiety compared with more mature students aged 35 and above (μ = 1.377, *p* < 0.001). Students' anxiety levels were also different based on locations where those from East Malaysia region displayed lower anxiety symptoms (μ = 1.386, *p* < 0.001) compared to other regions. Moving on, students' levels of anxiety were different based on risk to contagion level and ethnicity status. Surprisingly, those in the moderate risk area were experiencing higher anxiety symptoms (μ = 1.765, *p* < 0.05) compared with those in the high-risk area (μ = 1.608, *p* < 0.05).

Meanwhile, for DASS-D, depression levels were different based on age and location. Older students aged 35 years old and above were found to report lower levels of depression (μ = 1.500, *p* < 0.001) compared to younger students aged between 17 and 28 years old (μ = 1.667, *p* < 0.001; μ = 1.624, *p* < 0.001). We also found that students in East Malaysia region were experiencing lesser depression symptoms (μ = 1.681, *p* < 0.001) compared with students in other regions.

Three factors were found significant when comparing students' happiness levels. First, older students tend to be happier (29–34 years old: μ = 4.508, *p* < 0.001; 35 years old and above: μ = 4.590, *p* < 0.001) compared with younger students (19–22 years old: μ = 4.102, *p* < 0.001; 23–28 years old: μ = 4.172, *p* < 0.001). Second, students in the East Malaysia (μ = 4.383, *p* < 0.01) and Northern region (μ = 4.354, *p* < 0.01) tend to be happier than those in other regions. Third, Malay ethnicity recorded highest happiness levels (μ = 2.275, *p* < 0.001) compared with other ethnicities.

Meanwhile, for both work-to-family and family-to-work conflict, only location and ethnicity was found to contribute to significant differences in terms of levels. Specifically, students from the East Coast region recorded the highest W-FC (μ = 2.463, *p* < 0.01) and F-WC (μ = 2.321, *p* < 0.01) compared to other regions. Moreover Malay students recorded the highest for both W-FC (μ = 2.376, *p* < 0.01) and F-WC (μ = 2.173, *p* < 0.01) compared with other ethnicities.

### Risk and Protective Factors of Students' Level of Negative Emotional Symptoms During the Period of COVID-19 Lockdown

Multivariate logistic regression was performed using the Enter method to analyze students' risk factors to develop severe to extremely severe level of depression, anxiety, and stress symptoms. A summary of the results is presented in Table 7. Our overall model was significant, suggesting a good fit between data. A total of six predictors were tested: age, level of study, risk to contagion, happiness, work-to-family conflict, and family-to-work conflict. The dependent variables were clustered into two categories. “Zero” referring to a baseline group or cases with normal to moderate levels of negative emotional symptoms while “1” is the observed group referring to cases with severe to extremely severe levels of negative emotional symptoms. The results suggested that demographic factors of age, level of study, and risk of contagion did not predict the odds of occurrence severe or extremely severe stress, anxiety, and depression symptoms. Happiness was found as a protective factor, suggesting that a greater happiness score lowers the odds of experiencing severe to extreme stress (OR = 0.240, 95% CI: 0.180, 0.321), anxiety (OR = 0.336, 95% CI: 0.273, 0.414), and depression (OR = 0.121, 95% CI: 0.088, 0.165). Meanwhile, both work-to-family conflict and family-to-work conflict are found as risk factors for developing severe to extreme levels of different negative emotional symptoms. Detailed results suggest that a higher score of work-to-family conflict increases 1.45 times the odds of suffering from severe to extremely severe anxiety symptoms (OR = 1.453, 95% CI: 1.161, 1.818) while a greater score of family-to-work conflict increases the odds of experiencing severe to extremely severe stress and anxiety symptoms by 1.47 times (OR = 1.468, 95% CI: 1.109, 1.943) and 1.32 times (OR = 1.317, 95% CI: 1.059, 1.683) respectively.

**Table 7:** Predictors to severe stress (>25), anxiety (>14), and depression (>20) symptoms.

**Variable**	**DASS-S**		**DASS-A**		**DASS-D**	
	**OR (95% CI)**	***p***	**OR (95% CI)**	***p***	**OR (95% CI)**	***p***
**Age**	1.056 (0.740, 1.508)	0.763	0.782 (0.598, 1.023)	0.072	0.959 (0.692,1.328)	0.801
**Level of study**						
Diploma	0.577 (0.189, 1.757)	0.333	1.529 (0.792, 2.955)	0.207	1.826 (0.826, 4.038)	0.137
Master's degree	0.970 (0.362, 2.599)	0.952	0.642 (0.285, 1.447)	0.285	1.656 (0.700, 3.921)	0.251
Doctoral degree	0.594 (0.141, 2.504)	0.478	0.828 (0.290,2.363)	0.724	0.280 (0.063,1.239)	0.09
Others	1.010 (0.257, 3.968)	0.988	2.060 (0.807, 5.257)	0.131	0.747 (0.215, 2.603)	0.647
Bachelor's degree	Reference	1.00	Reference	1.00	Reference	1.00
**Risk of contagion**						
High risk	3.572 (0.759, 16.815)	0.107	0.962 (0.437.2.120)	0.923	0.944 (0.362, 2.460)	0.906
Moderate risk	4.194 (0.807, 21.790)	0.088	1.922 (0.797, 4.632)	0.146	0.844 (0.248, 2.506)	0.760
Low risk	Reference	1.00	Reference	1.00	Reference	1.00
**Happiness**	0.240^***^ (0.180, 0.321)	0.000	0.336^***^ (0.273, 0.414)	0.000	0.121^***^ (0.088, 0.165)	0.000
**Work-family conflict**	1.183 (0.888, 1.577)	0.250	1.453^***^ (1.161, 1.818)	0.001	1.168 (0.894, 1.525)	0.254
**Family-work conflict**	1.468^**^ (1.109, 1.943)	0.007	1.317^*^ (1.059, 1.638)	0.013	1.172 (0.900,1.527)	0.239

* p < 0.05;

** p < 0.01;

*** *p < 0.001*.

### The Link Between Negative Emotional Symptoms, Happiness, and Work-Family Constructs

**Figure 1:**
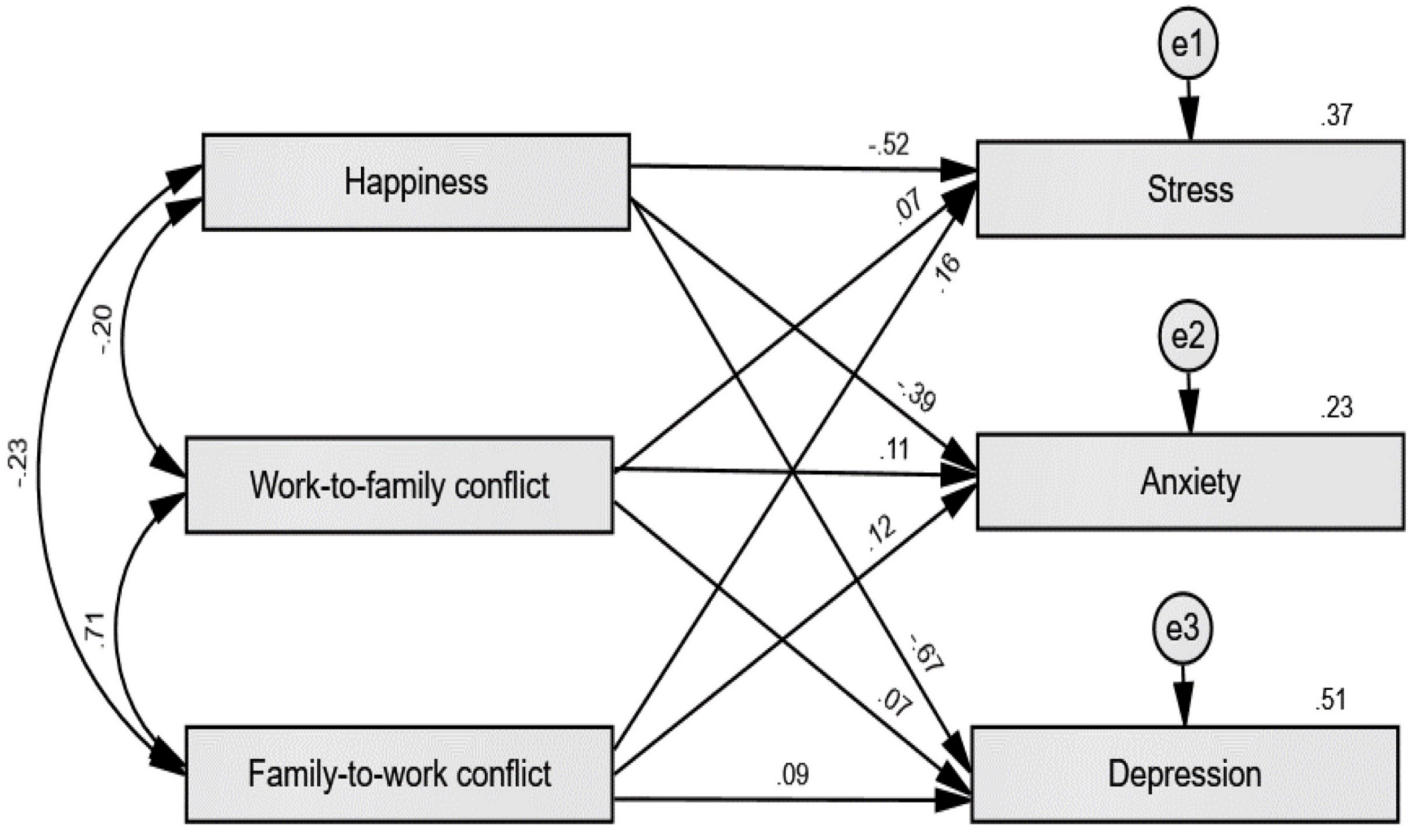
Path analysis model for the significant effects between mental health, happiness, and work-family conflict (*N* = 1,005).

To further examine the linkages between constructs in this study, we performed path analysis a subset of SEM technique using AMOS software which overall finding is illustrated in Figure 1. Result suggests that all paths in this study are found significant. Mainly, the increase in happiness level is linked to lower stress (β = −0.521, *p* < 0.001), anxiety (β = −0.387, *p* < 0.001), and depression (β = −0.666, *p* < 0.001) symptoms. Meanwhile, both high work-to-family and family-to-work conflicts are linked to higher stress (W-FC: β = 0.074, *p* = 0.036; F-WC: β = 0.162, *p* < 0.001), anxiety (W-FC: β = 0.110, *p* = 0.005; F-WC: β = 0.115, *p* = 0.004), and depression symptoms (W-FC: β = 0.071, *p* = 0.024; F-WC: β = 0.094, *p* = 0.003).

## Discussion

### Students' Levels of Negative Emotional Symptoms, Happiness, and Work-Family Conflict During the Period of COVID-19 Lockdown

The implementation of the first MCO in Malaysia was generally found to be effective for curbing the spread of COVID-19; however, less is known on how it affected the psychological outcomes of university students. In terms of negative emotional symptoms, it appears that the majority of students experience normal level of stress symptoms. However, a major cause of concern was that nearly a quarter of students experience severe to extremely severe symptoms of anxiety and depression. Notably, nearly 40% of the students experience moderate to extremely severe symptoms of anxiety and depression. In comparison with a study conducted immediately after the COVID-19 outbreak in China, 16.5% reported moderate to severe depressive symptoms and 28.8% reported moderate to severe anxiety symptoms (24). Our findings to some extent are consistent with other studies that reported that outbreaks may trigger negative emotions either for COVID-19 (25) and SARS outbreaks (26, 27). This highlights the urgent need to cater to the mental health needs of the students to prevent further escalation without appropriate early interventions. Despite the alarming level of negative emotional symptoms, half of the students rated their happiness level as “rather happy” or “very happy.” Our findings highlight further evidence that happiness can be maintained despite the fluctuating nature of our positive and negative mood (17).

This study also extends the understanding of students' state of work-family conflict during the period of MCO. Despite the lack of discussion on this notion among this population (11), our findings established compelling evidence that students are likewise experiencing interference of work and life conflict alike the working population. Work-family conflict refers to pressure from one area of life that negatively affects the other (19). An alarming rate of 50.4 and 39.2% of our total respondents were found to be experiencing high to very high levels of W-FC and F-WC which is significantly higher than average levels reported in the past research (11). This signifies that unconducive environment due to MCO carries certain negative implications to the students' everyday life. Having to stay at home with family members or to be confined in restricted university accommodations for a longer period may contribute to a different obstacle in their everyday life. Besides, the lack of resources and immense changes in terms of assessment might contribute to the conflict occurrences. Findings suggest that more students are experiencing higher work-to-family conflict compared with family-to-work conflict. In line with past research (11, 20), work behavior inflicts more damage to the family aspect, rather than the vice versa. Hence, engaging and spending time in academic-related activities such as online learning, assignment, and other related activities are perceived as a greater source of conflict during the MCO period.

### Differences in Students' Level of Negative Emotional Symptoms, Happiness, and Work-Family Conflict Based on Demographic Factors

Further analysis indicated that the level of stress, anxiety, and depression were significantly different according to age and geographic location. Younger students experienced more stress, anxiety, and depression symptoms compared with older ones. Research on the wider youth population conducted during the COVID-19 outbreak found younger college and school students reported higher levels of psychological distress compared with older students (8). Our study, however, does not record statistically significant different levels of symptoms according to study level (i.e., diploma, degree, masters, etc.) with consideration that the proportion of undergraduate degree students far exceeded the others. Those in central locations were more depressed, while those in the east coast region were more stressed and anxious. While the risk of contagion varies within specific locations in the central region, it can be said that the central region in Malaysia is more densely populated compared with other regions with more number of positive COVID-19 cases reported in the central region.

There is also a significant difference and higher stress and anxiety symptoms among private university students compared with public university students. In general, most public universities in Malaysia are equipped with more comprehensive facilities in offering support to the students. Undeniably, attention to all students regardless of background is imperative, and our data highlights the need for more support for those in the private universities. Anxiety symptoms also differed according to the risk of contagion of the different locations. Unsurprisingly, much lower symptoms were recorded in low-risk regions or the “green zones.” However, slightly higher anxiety symptoms were recorded in moderate-risk regions compared with high-risk regions. A possible explanation for this is that the high-risk region may already be on red alert whereas moderate-risk regions may subdue its possible negative implications causing uncertainties. In essence, anxiety may also develop due to uncertainties of possible threats which compromises our ability to avoid or mitigate its negative implications (28).

Our data also shows that older students tend to be happier than younger students during the COVID-19 lockdown. Previous studies have reported that global happiness of university students follows an inverted parabola pattern in which happiness gradually increases as students aged but a sudden drop is observed with older students (29–31). It should be highlighted that nearly 90% of our respondents were between 17 and 28 years old that was consistent with the inverted parabola pattern. On the other hand, our findings show that the increment continues for older students despite the significantly underrepresented group of older students. Moreover, students' happiness levels also varied based on location. Those in the central region recorded lowest happiness level, followed by those who are currently situated in the east coast and southern regions. These findings suggest that students located at the central region were least happy compared with the rest of the population, and these might be due to several plausible explanations. At times where this study was conducted, the central region was the most badly affected area due to COVID-19 with more than half of the subregions declared as “red” or high-risk zones. Dealing with very strict rules and such limited access to facilities might contribute to their slightly lower happiness level compared with the rest.

Meanwhile, there was a significant difference in terms of means level based on the type of university, location, and ethnicity for the experience of work-family conflict. Public university students, in particular, were found to record higher W-FC level compared with those from private universities. Besides, the finding indicates that those in the east coast and southern region to report a higher level of W-FC compared with the rest. Malay ethnicity experienced higher W-FC levels compared with Indian and other ethnic groups. While it is difficult to provide a meaningful explanation to some of these findings, these patterns give some valuable insight on conflict experience based on demographic factors. It was argued that students who received greater support from the university and with higher satisfaction were likely to experience lower between domain conflict (32). This signals that gaps might exist between strategy and level of support implementation given by public and private university to students during the MCO period which may contribute to such differences. It highlights a pivotal point for policy makers or researchers to focus on a specific ethnic group, Malay which is found more vulnerable to experiencing higher between domains conflict.

### Risk and Protective Factors of Students' Level of Negative Emotional Symptoms During the Period of COVID-19 Lockdown

Our findings indicated that students with higher levels of happiness have decreased probability to have severe or extremely severe symptoms of stress, anxiety, and depression. Such a finding was also aligned in the path analysis, whereby we found evidence on the negative linear relationship between happiness with the three mental health constructs. Likewise, a large-scale cross-sectional study on 2,383 university students in Korea whereby happiness is associated with lower risk of depression symptoms (33). For decades, the field of clinical psychology has primarily focused on problems and deficits. In recent years, however, positive factors of psychological well-being pioneered by Martin Seligman have been starting to receive wide attention among researchers (34). In essence, our findings build on the fact that while negative symptoms may precipitate negative emotional symptoms, positive aspects such as happiness can be a protective factor for negative emotional symptoms among university students. Importantly, our evidence establishes a strong link between low happiness level with higher depression level, which suggests the importance of improving student's subjective experience as one of the focal points to improve their mental health. Consistent with this, the directional nature can be two ways as once highlighted by Richard Layard, the coeditor of the World Happiness Report; “Better treatment for mental health would improve happiness directly; and improving happiness in other ways would reduce the frequency of mental illness” (35).

On the negative note, this study identifies work-family conflict and family-work conflict as potential risk factors for developing severe or extremely severe anxiety and stress symptoms. This finding aligns with our path analysis result which suggested positive links between work-family conflict with stress, anxiety, and depression. In line with a previous study conducted in Minia, Upper Egypt, W-FC was associated with an increase in the probability of developing severe anxiety symptoms (36). Moreover, the likeliness of students developing severe stress and anxiety is higher following the increasing score of F-WC. Following the concept of work-family conflict as a form of inter-role conflicts, this study provides support from the student's context that work-family and family-work conflicts may contribute to stress and anxiety. It also stresses the potential risk of students' everyday environment and their life integration practice during unprecedented times of COVID-19 as an area that needs serious attention. It is evident from past research that overall educational experience plays a great role in eliciting between domains conflict (32), henceforth monitoring such occurrence will reduce the risk of negative emotional symptoms among students' population.

## Implications and Recommendations

In this section, implication and recommendation of this study will be emphasized by using the Strengths, Weaknesses, Opportunities, and Threats (SWOT) strategies.

### Strengths

Most institutions have their own vision planning and during this time, challenges should complement strategic planning based on current resources. Most universities do have facilities to manage financial structure, academic curriculum, and psychosocial support. Therefore, they need a strategic plan that can benefit all parties involved in universities. Enhance the role of psychosocial support not only among students but also their families and all university staff to ease the burden of public health care. Online psychological interventions such as mindfulness-based therapy and cognitive behavior therapy were found to be helpful during the COVID-19 pandemic (37). University management could offer structured support systems in the areas of academic, finance, and counseling (both career and psychological health). Hence, it is of prominent importance that universities establish guidelines for virtual services that include all systems such as counseling, lecture, enrolment, and industrial collaboration. If online counseling is to be implemented by universities, a proper guideline is also needed considering privacy, confidentiality, and professional ethics besides the emphasis on the accessibility and availability of technology for students from varied locations.

### Weaknesses

Clearly, enrolment of domestic and international students will be reduced and on-campus tuition will not begin until 2021 resulting to the financial structure being affected. University management, lecturers, and research supervisors should change old practice to suit new norms together to ease the needs of students. This aspect is essential not only to understand academic challenges but also work and family conflict that may be experienced by students while also recognizing the challenges they face such as work from home, children's challenges, and other factors. These two issues will continue to be part of weaknesses if people are not flexible in adjusting to changes that are deemed as necessary.

### Opportunities

Due to the above weaknesses, vision planning will coexist and complement strategic planning. For instance, private and small colleges and universities can collaborate with other institutions. The academic structure may combine the best of both direct and online learning approaches. The recruitment activities throughout the year will allow applicants more flexibility in the selection and enrolment of colleges and universities. New business models and financing options will bring stability to the “bottom line.” All universities should be encouraged for cooperation rather than competition to ensure both parties (i.e., universities and ministries; students and family) can benefit behind this pandemic. Replace competition with collaboration between colleges, private universities, and governments that have had to postpone operations or even shut down due to declining enrolment and student income. Universities that have the potential to be financially viable can offer intellectual or material collation so that the impact of the virus is not to cease the operation of educational institutions but to revitalize and strengthen the education system forward.

In terms of offering psychosocial services and support such as assessment and intervention (counseling, psychology, and psychiatry), professionals in these fields need to not only double their efforts in the traditional practice locations but also facilitate the use of virtual technology beyond logistics restrictions to provide appropriate services to students irrespective of their physical locations. This is even more imperative considering the high number of referral cases received by the public health services for the assessment and interventions of psychological issues in Malaysia (38). Programs utilizing psychologically positive methods can also be implemented for those affected by this COVID-19 pandemic to enhance aspects of happiness, gratitude, and emotional regulation. For students with diagnosis of mental illness or even with early signs of disorders, serious attention should be given so that the consequence will not worsen.

### Threats

COVID-19 pandemic accelerates the end of the traditional semester-based system for college enrolment, progress, and graduation. Some colleges and universities may be forced to close down. If the academic year is restructured, then the recruitment year must be restructured. An effective method of communication is important to ensure future students, graduates, and their parents are well-informed about any changes to be made. New business models and financing options shall be introduced to encourage students to continue their education without adding more burden to their families. The amount of annual/semester fees and other required fees should be reconsidered by the universities accordingly. Economic vulnerabilities may be one of the reasons for students' reluctance to seek help if they experience COVID-19 symptoms. These imply a need for involving the university clinics and health services as local health gatekeepers who are the first point of detecting and reporting of suspected COVID-19 cases (39), as well as a channel where accurate information regarding COVID-19, protective equipment, and intervention packages can be delivered (40). Having COVID-19 testing centers in the universities are also recommended (39).

In sum, the psychological impact of the COVID-19 pandemic allows fresh opportunities for all parties to improve current financial management weaknesses, advance higher education curricula, enhance online learning opportunities, and most importantly improve communication between students, families, and universities to collaborate for the sake of good mental health and future prospect.

## Limitations and Future Research

This study has several limitations. In view of the MCO restrictions, we employed the non-probability snowball sampling method, therefore the findings do not reflect the overall patterns of negative emotional symptoms, happiness, and work-life balance and are not generalizable to the wide university student population in Malaysia. Moreover, our study also did not investigate possible confounders such as electronic device availability, Internet accessibility, premorbid personality, and coping style that could have an influence toward the study results. In addition, since this study was conducted during specific MCO period, thus findings are subjected to the unique circumstance of when this study took place. It cannot however be used to understand post-MCO period behavior. Therefore, future researchers might want to conduct studies investigating fluctuation in terms of emotional and behavioral symptoms between the lockdown transition periods.

## Data Availability Statement

The raw data supporting the conclusions of this article will be made available by the authors, without undue reservation.

## Ethics Statement

The studies involving human participants were reviewed and approved by Faculty of Arts and Social Sciences, University of Nottingham Malaysia Campus ethics committee (Approval number: FASS2020-0008/DOAP/SKH). The patients/participants provided their written informed consent to participate in this study.

## Author Contributions

WMAWMY and SKZB contributed to the conception of the study, contributed significantly to analysis, manuscript preparation, performed the data analyses, and wrote the manuscript draft. SAP and FM helped modify the manuscript. WMAWMY, SKZB, SAP, and FM contributed to the interpretation and discussion of the results of the analysis. All authors contributed to the article and approved the submitted version.

## Conflict of Interest

The authors declare that the research was conducted in the absence of any commercial or financial relationships that could be construed as a potential conflict of interest.
